# Drivers of farmer-managed natural regeneration in the Sahel. Lessons for restoration

**DOI:** 10.1038/s41598-020-70746-z

**Published:** 2020-09-14

**Authors:** Madelon Lohbeck, Peggy Albers, Laetitia E. Boels, Frans Bongers, Samuel Morel, Fergus Sinclair, Bertin Takoutsing, Tor-Gunnar Vågen, Leigh A. Winowiecki, Emilie Smith-Dumont

**Affiliations:** 1 World Agroforestry (ICRAF), P.O. Box 30677-00100, Nairobi, Kenya; 2 Forest Ecology and Forest Management Group, Wageningen University, P.O. Box 47, Wageningen, The Netherlands; 3 School of Natural Sciences, Bangor University, Wales, UK; 4 World Agroforestry, P.O. Box 16317, Yaoundé, Cameroon; 5 Soil Geography and Landscape Group, Wageningen University, P.O. Box 47, Wageningen, The Netherlands

**Keywords:** Agroecology, Ecosystem ecology, Ecosystem services, Restoration ecology, Agroecology, Ecosystem ecology, Ecosystem services, Restoration ecology

## Abstract

Farmer-managed natural regeneration (FMNR) is being promoted for restoration beyond its original range in the Sahel. FMNR involves farmers selecting and managing natural regeneration on their fields, while keeping them under the primary function of agricultural production. However, little is known about what regenerates in different contexts, even though this underlies potential restoration impact. Here we assess how human impact, land degradation and dispersal limitation affect structural and functional properties of regeneration across 316 plots in agroforestry parklands of Ghana and Burkina Faso. We found that intensity of land use (grazing and agricultural practices) and dispersal limitation inhibited regeneration, while land degradation did not. Functional composition of regenerating communities shifted towards shorter statured, small-seeded and conservative strategies with intensity of land use. We conclude that the presence of trees of desired species in the vicinity is a precondition for successfully implementing FMNR for restoration, and that regeneration needs to be protected from grazing. Assessment of regeneration potential is imperative for scaling out FMNR and where natural regeneration will be insufficient to achieve restoration targets, FMNR needs to be complemented with tree planting.

## Introduction

The UN general assembly recently declared 2021–2030 as the decade of ecosystem restoration. It aims to massively scale up the restoration of degraded and destroyed ecosystems to fight the climate crisis and enhance food security, water supply and biodiversity. Natural regeneration is considered a promising solution to restoration^1^; it is cheap, has a low labour requirement and a great potential to restore carbon^2^, biodiversity^3^ and other ecosystem functions^4^. Most knowledge about natural regeneration comes from successional studies where agricultural fields are abandoned and the natural ecosystem is allowed to regenerate^5^. This is not feasible for much degraded land in agricultural landscapes that farmers depend on for their livelihood. Abandoning fields for restoring functions, even if they are functions that farmers depend on, is often not an option because of land scarcity^6^. In such cases farmer-managed natural regeneration (FMNR) can be effective, for example, in the agroforestry parklands of Niger where more than 3 million hectares have been revegetated since the 1980s^7^. FMNR involves farmers nurturing natural woody regeneration on their fields, while keeping them under the primary function of agricultural production (see Box 1). Examples from Niger and more recently also from Burkina Faso, Mali, Senegal and Ethiopia show that FMNR can reverse the loss of tree cover and diversity in dryland systems^8^, increase soil carbon^9^, crop yield and raise household income^10,11^.

The ultimate composition of regenerated vegetation depends on what species regenerate, their survival and the specific land and tree management practices that farmers employ, although these have been sparsely documented in the literature. To address this knowledge gap, we take a traits-based approach to understanding regeneration dynamics. Traits-based approaches make use of species functional traits with the aim to mechanistically link land-use with species performance and ecosystem function^12,13^. For regeneration to be found on farmers’ fields it first needs to arrive (by seed) or be present in the soil (by seed stock or root stock), then it needs to germinate (from seed) or resprout (from rootstock) to grow into a seedling or sprout, after which regeneration needs to grow and survive field conditions (which may be characterized by environmental stress and frequent disturbance) and be of enough interest to the farmer to be protected and not removed. In functional ecology these steps are described as environmental filters where functional traits may explain species' ability to pass through the filters^14^. Functional traits may therefore predict species performance in a restoration context^15^. In this study we evaluate three main environmental filters that may hamper or promote regeneration in the context of FMNR leading to a mechanistic understanding of regeneration. These are: the abundance and proximity of seed sources to reveal possible effects of dispersal limitation, land degradation as a proxy for environmental stress, and human impact on the land to indicate frequent disturbances. To date it is largely unknown how dispersal limitation, land degradation and anthropogenic drivers affect regeneration dynamics in the context of natural regeneration for restoration. This is crucial for identifying the potential of FMNR as a scalable restoration technique, where and for whom it is likely to be a useful option^16^, and thereby its usefulness in the ecosystem restoration decade.

We studied seven whole plant traits, two reproductive traits, and seven leaf traits that determine the carbon, water and nutrient balance of plants and are expected to determine species success under differing conditions of dispersal limitation, stress and disturbance. For instance, small seeded species can disperse long distances by wind making them effective colonizers when seed sources are far^17^, while large seeded species are better able to survive harsh and degraded conditions^18^. Resprouting helps a species persist under moderate levels of disturbances like fire and herbivory^19^. Invasive properties help species to proliferate where soil fertility is low but may, in turn, have a negative effect on the crops and on soil conditions causing farmers to eliminate them^20,21^. In contrast, farmers may promote N_2_-fixers for their beneficial effects on the soil^22^, and tall species for economic value. Deciduous species better survive dry and degraded conditions^23^. Species with acquisitive leaf traits (large leaves, high specific leaf area, chlorophyll contents and instantaneous chlorophyll fluorescence) grow fast but need optimal light and soil conditions while species with conservative leaf traits (high leaf dry matter content, leaf density and leaf thickness) are able to persist under harsh degraded environments and frequent disturbance^24^.

In this study we use data from agroforestry parklands in Burkina Faso and Ghana where FMNR is being practiced (See also Supplementary Fig. 1). We aimed to identify the effects of dispersal limitation, human impact and land degradation on regeneration density, diversity and functional traits.

We hypothesize that (1) **dispersal limitation**; the density and diversity of natural regeneration is positively influenced by the closeness to forest reserves and by the abundance of adult trees. We expect more regeneration from seed and larger seeded species closer to reserves and with more adult trees while further away regeneration through resprouting may be more prevalent. (2) **land degradation**; highly degraded soils, characterized by erosion and low soil fertility, create harsh conditions that negatively affect regeneration density and diversity and favours conservative functional strategies, more resprouts, invasive species and N_2_ fixers. (3) **human impact**; the intensity of field management practices (impact of grazing, agriculture and fire) has a negative effect on regeneration density and diversity. We expect specific effects of field management intensity on the functional properties of the regenerating community where grazing and fire may promote conservative functional strategies, while with intensity of agricultural use N_2_ fixers and tall species are more likely to be promoted by farmers because these are less likely to compete with crops.

**Figure 1:**
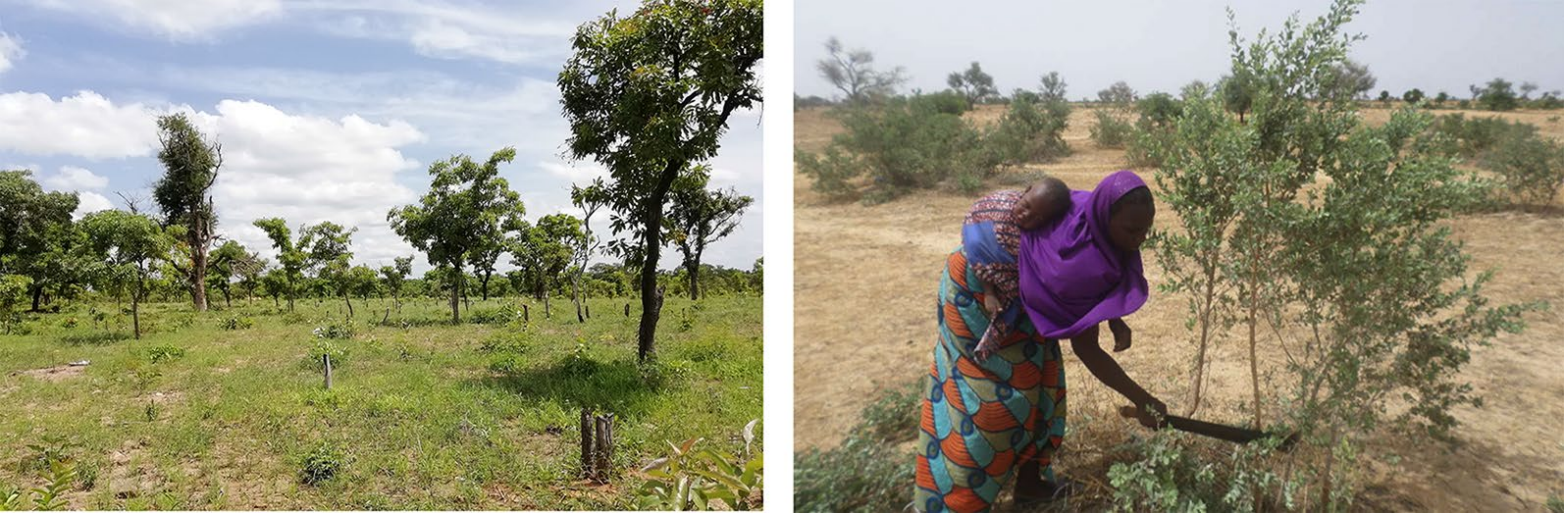
(**a**) Sahelian agroforestry parkland in the study region in Ghana (photo by P. Albers). (**b**) Farmer removing side stems from *Guiera senegalensis* to enhance its growth (photo by P. Savadogo/ ICRAF).

Box 1: What is FMNR? Farmer-managed natural regeneration is a way to restore farmland. In FMNR farmers select and promote regeneration of trees and shrubs on farmland that has come up through natural regeneration, while keeping the land under the primary function of agricultural production (Fig. 1). Natural regeneration occurs through resprouting tree stumps, root stock or from recruiting seeds that are present in the soil or get dispersed into the field. Farmers promote regeneration through pruning, mulching and active protection (Fig. 1b). Since tree planting is not needed, FMNR is relatively cheap, accessible and may have higher tree survival rates. Restoration benefits from FMNR have been demonstrated mainly by studies carried out in West African parklands. However little is known about how land management, land degradation and dispersal limitation affect natural regeneration across farmlands. This understanding is urgently needed to optimize strategies for scaling out FMNR beyond its countries of origin.

## Results

### Characterizing the study landscapes

The study landscapes consisted of a mosaic of land uses including crop fields (41%), fallows (41%), grassland (7%) and forest or woodlands (13%). Plots varied in their human impacts, environmental drivers and level of degradation. In terms of human impact 49% of the plots had agricultural activities, in 64% grazing took place and 54% showed signs of fire use, though their intensities differ across land uses (Supplementary Fig. 2) and across the two sites (Supplementary Fig. 3). 82% of the plot were eroded and over 99% had critically low levels of topsoil nitrogen (< 0.2%). The median tree density is 30 trees per hectare and 79% of the plots had at least one tree. See Supplementary Fig. 2 for variation in variables studied across land uses.

**Figure 2:**
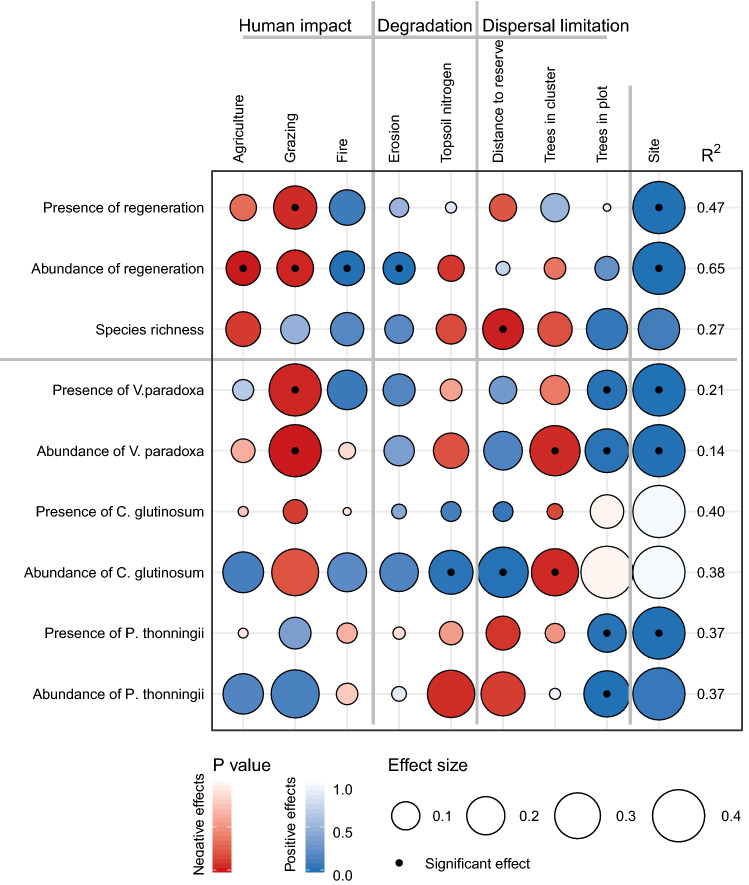
Effects of drivers (columns) on the structural properties of the regenerating community (rows), as tested with separate generalised linear models. Given are the effect sizes (standardized beta, size of the circle). Blue and red indicate positive and negative effects while the shading indicates the P value, significant effects (P < 0.05) are indicated with a black dot in the circle. Model fit estimated by the Nagelkerke R^2^ is given for each model. Drivers of regeneration are categorized into human impact, land degradation and dispersal limitation. Site (Kayoro in Ghana and Seloghin in Burkina Faso) is included as a fixed effect, here effect size indicates how different is Seloghin compared to Kayoro, and effects are given to a maximum effect of 0.4 for visibility purposes, see Supplementary Table 1 for actual values. Structural properties include presence of regeneration, the abundance of regeneration and rarefied species richness as well as the presence and abundance of the three most important regenerating species; *Vitellaria paradoxa, Combretum glutinosum, Piliostigma thonningii*. For presence all data are used (N = 318) for abundance of seedlings and species richness the subset of plots where regeneration is present was used (N = 152). See Supplementary Table 1 for exact values.

The median density of regeneration was 0 and regeneration was present in 48% of our plots, more in Burkina Faso (75%) than in Ghana (21%). Burkina Faso also had higher abundance and richness of regeneration (Fig. 2). 1,238 trees and 679 regenerating individuals were recorded belonging to a total of 86 species, 68 species across the tree communities and 50 species across the regeneration communities. The single most common species was *Vitellaria paradoxa* representing 38% of all trees and 12% of all regeneration. In the tree community *Vitellaria* is followed by *Anogeissus leiocarpus* (10%) and by *Lannea acida* (5%) while in the regeneration community *Vitellaria* is followed by *Combretum glutinosum* (12%) and *Piliostigma thonningi* (11%) (Supplementary Figs. 4 and 5).

### Factors influencing structural and functional properties of regeneration

**Figure 3:**
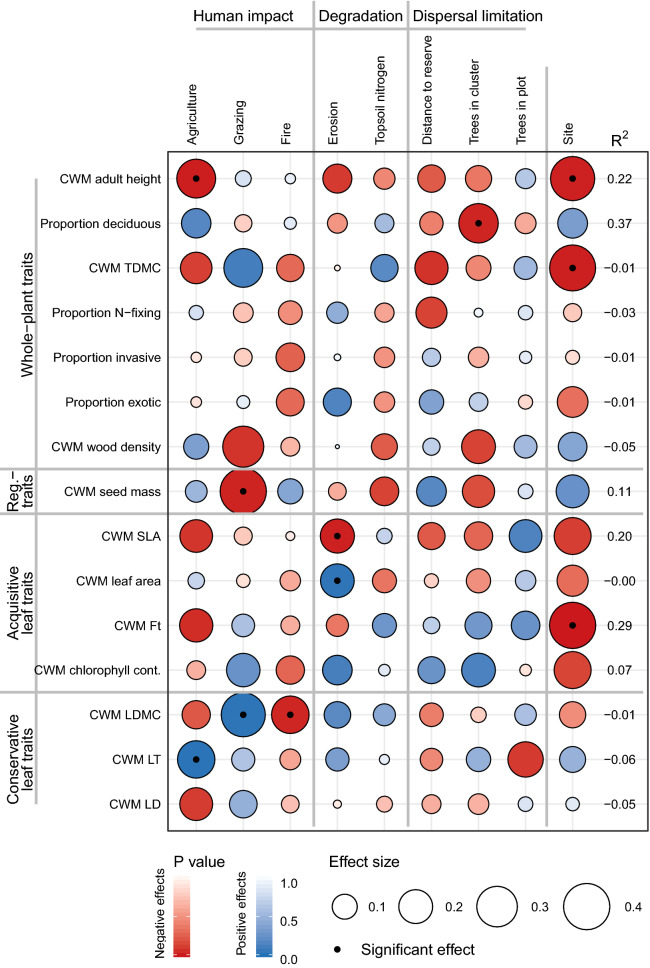
Effects of drivers (columns) on the functional properties of the regenerating community (rows), as tested with separate generalised linear models (N = 152). Given are the effect sizes (standardized beta, size of the circle). Blue and red indicate positive and negative effects while the shading indicates the P value, significant effects (P < 0.05) are indicated with a black dot in the circle. Model fit in estimated by the Nagelkerke R^2^ is given for each model. Drivers of regeneration are categorized into human impact, land degradation and dispersal limitation. Site (Kayoro in Ghana and Seloghin in Burkina Faso) is included as a fixed effect, here effect size indicates how different is Seloghin compared to Kayoro, and effects are given to a maximum effect of 0.4 for visibility purposes, see Supplementary Table 1 for actual values. Functional properties represent the community-weighted means based on whole-plant traits, regenerative traits, acquisitive leaf traits and conservative leaf traits. See Supplementary Table 1 for exact values.

Of the 25 models tested, 15 regeneration variables were significantly explained by at least one driver (site is not considered a driver). Structural properties were better explained (8 models out of 9; Fig. 2) than functional properties (7 out of 15; Fig. 3).

The presence of *C. glutinosum*, and the community-weighted mean of twig dry matter contents, N_2_ fixing, invasiveness, exotics, instantaneous chlorophyll fluorescence, chlorophyll content and leaf density were not explained by any driver, while the community-weighted mean of resprouting capacity could not be tested because there was no variation across communities (all species encountered had the potential to resprout). Of the 15 variables that were explained by our model, in eight cases human impact explained changes in the regenerating community, in four cases the level of land degradation and in seven cases dispersal limitation had a significant effect (Figs. 2, 3). In terms of the structural characteristics of the regenerating communities we found that grazing was negatively associated with presence of regeneration (β = -0.26, P = 0.045) and its abundance (β = -0.18, P = 0.027) of all species and also of presence (β = -0.46 P = 0.031) and abundance (β = -0.74 P = 0.004) of *V. paradoxa* specifically. Agriculture negatively influenced regeneration abundance (β = -0.16 P = 0.005) while fire had a positive effect (β = 0.16, P = 0.003). Erosion increased the abundance of regeneration (β = 0.13 P = 0.004) while topsoil nitrogen increased the abundance of *C. glutinosum* (β = 0.27 P = 0.022). Closer to forest reserves regeneration had higher species richness (β = -0.23 P = 0.014) while the abundance of *C. glutinosum* decreased (β = 0.37 P = 0.009). The abundance of conspecific trees in the cluster decreased regeneration abundance of *V. paradoxa* (β = -0.37 P = 0.046) and *C. glutinosum* (β = -0.32 P = 0.040) while the abundance of conspecific trees in the plot increased *V. paradoxa* presence (β = 0.21 P 0.017) and abundance (β = 0.27 P = 0.019) as well as *P. thonningii* presence (β = 0.20 P = 0.017) and abundance (β = 0.31 P < 0.001) (Fig. 2).

In terms of the functional characteristics of the regenerating community we found that with more intense use for agriculture the regenerating species in the plot tended to be of shorter adult stature (β = -0.27 P = 0.009) and have thicker leaves (β = 0.25 P = 0.021). Grazing resulted in lower seed mass (β = -0.41 P = 0.023) and higher leaf dry matter contents (β = 0.36 P = 0.030). Fire reduced the leaf dry matter content (β = -0.25 P = 0.032). Land degradation in the form of erosion decreased the specific leaf area (β = -0.20 P = 0.020) and increase the leaf area of seedlings (β = 0.20 P = 0.035) while soil nitrogen had no effect on functional properties. The number of trees in the cluster had a negative influence on the proportion of deciduous species (β = -0.28 P = 0.028) in the regenerating community (Fig. 3).

## Discussion

With the eyes of the world towards natural regeneration as an effective and cheap solution to restoring degraded lands, it is vital to understand under what conditions natural regeneration takes place and what the structural and functional characteristics are of the regenerating community. In this study we draw from functional ecology to analyse the effects of human impact (disturbance and farmers preferences), land degradation (stress) and dispersal limitation (seed source availability) on regenerating communities in agricultural landscapes where farmer-managed natural regeneration takes place. While assessing nine structural and 16 functional properties of regenerating communities we found that human impact is the most important factor influencing regeneration (affecting 8 variables), closely followed by dispersal limitation (affecting 7 variables), while land degradation was less important (affecting 4 variables). All drivers affected both structural and functional properties of regenerating communities, although different drivers affected different variables. In general stress and disturbance created communities with short-statured species and conservative traits that specialize to persist, though also opposite shifts were found indicating the opportunistic and acquisitive strategy of newly colonized regeneration. Useful species *Vitellaria paradoxa* and *Pilliostigma thonningii* are strongly dispersal limited. Effects of dispersal limitation on structural properties stress the importance of trees and forests as sources of diversity and warrant potential limitations of FMNR beyond tree-dense agroforestry parklands.

Structural properties of regenerating communities were influenced by disturbance, stress and by dispersal limitation. In terms of human impact we found that grazing was the most important factor negatively influencing presence and abundance of regeneration (Fig. 2) as well as the presence and abundance of *V. paradoxa*. It is well known that herbivory damages plants and can inhibit recruitment^25,26^. Indeed, livestock grazing is considered harmful for forest regeneration in many parts of the West African drylands^27^. Also intensity of use for agriculture reduced regeneration abundance. High impact of agriculture is exclusive to crop fields (Supplementary Fig. 3) where crop production is the primary purpose. Removal of undesired regeneration is inherent to the practice of FMNR, and this will be especially apparent in active crop fields where the farmer will try to avoid competition with crops. The impact of fire increases the abundance of regeneration across our sites, which was against expectations as fire is a severe form of disturbance which can kill regeneration e.g.^28,29^. In Sahelian parklands two types of fire are commonly distinguished; early fires (occurring early in the dry season) and late fires (occurring late in the dry season) when the vegetation is dry. Early fire may not inhibit regeneration through resprouting^30^ while late fire does have the capacity to destroy regeneration and even adult trees on the long term^31^. In this study we cannot distinguish between early and late fires although the intensity of fire (scale 0–3) probably scores higher with late fires. All species encountered had the ability to resprout, so fire may have caused dieback without mortality^32^. Vitellaria has evolved the property of cryptogeal germination, where shoots stay underground for months, which is probably an adaptation to fire^33^. This adaptation may explain its success regenerating in burned crop fields and fallows^34^ although in this study we found no effects of fire on presence and abundance of Vitellaria*.*

Land degradation is a problem in the study region given that 82% of the plots show signs of erosion, and over 99% of the plots had topsoil nitrogen below the critical threshold of 0.2% needed to sustain agriculture^35,36^. We found no evidence that degradation limited regeneration presence and abundance, probably because these soils are naturally nutrient poor^37^, and species that naturally regenerate here are adapted to such conditions. Other studies did demonstrate that degraded soils can hamper recruitment, growth and survival^38^. Surprisingly, regeneration abundance was higher on more eroded plots, which suggests proliferation of degradation-adapted species. A more detailed look at the 4^th^ and 5^th^ most abundant regenerating species; *Combretum nigricans* and *Guiera senegalensis* revealed that these were associated with degraded soils. *C. nigricans* was negatively influenced by soil nitrogen and *G. senegalensis* was positively influenced by erosion severity and negatively by soil nitrogen. Both species belong to the family Combretaceae, and 'combretisation' is considered an indicator of degradation^39^. Contrary to this, *Combretum glutinosum*, the second most abundant regenerating species, is associated with increased topsoil nitrogen (Fig. 2). Although nitrogen is an important nutrient limiting plant performance, in line with the 'combretisation'-theory, we had also expected this species to be associated with degradation.

Dispersal limitation was the most important factor influencing the structural properties of regeneration. Regenerating communities tended to be more species-rich when they are located closer to forest reserves, highlighting the importance of forest reserves as sources for biodiversity. It shows that at least some species are hampered in their dispersal from the reserve to the cultivated areas, possibly they depend on animal dispersers (like bats, birds and rodents) that are more restricted to the forest^28^. For the two common locally valuable species *V. paradoxa* and *P. thonningii* their presence and abundance depended on the abundance of conspecific trees in the same plot, which is an indicator of dispersal limitation and suggests that these species, in these plots, mostly regenerate from seed. Indeed *V. paradoxa* disperses through barochory, meaning that seeds spread mainly by falling from the tree^40^. Effects of dispersal limitation are in line with previous research in the Sahel showing that the occurrence of seedlings seemed to depend on the presence of adults of that same species in the same field^26^.

Human impact, land degradation and environmental factors all directionally shifted the functional composition of regenerating communities, though human impact was the strongest factor. Disturbance and selection through human activities generally shifted regenerating communities towards shorter vegetation with smaller seeds and more conservative traits. Land degradation affected only leaf traits, while the dispersal limitation affected one whole-plant trait. Here we go into more detail on the specific patterns and underlying mechanisms. A shift towards shorter adult stature with more intense agricultural use and grazing indicates a replacement of tall tree species by more shrubby species, likely to be partially driven by the shrubs *P. thonningii* and *C. glutinosum* (Fig. 2). These species have a strong capacity to resprout after cutting and grazing^26^, allowing them to resist frequent disturbance. With more trees in the wider surroundings of the regenerating community (cluster) we found less individuals with a deciduous leaf habit. The ability to shed leaves in the dry season increases survival^23^ and our finding may indicate that the abundance of trees reduces drought conditions for seedlings, allowing also evergreen species to survive.

Conservative functional traits help plants to deal with stresses, like mechanical disturbance and drought^24^. We therefore expected more conservative traits with the intensity of human impact and with land degradation. This hypothesis was confirmed for agriculture increasing leaf thickness and for grazing increasing leaf dry matter content. Fire instead reduces leaf dry matter content, indicating a more acquisitive functional strategy, and a reduced flammability of the leaves^41^. In terms of land degradation we found no shifts in functional composition with topsoil nitrogen, while erosion had contrasting effects on the acquisitive-conservative spectrum; erosion reduced specific leaf area and increased leaf area (both acquisitive leaf traits). Contrasting effects of disturbance on community composition were also found in the Caatinga dry forest in Brazil; saplings shifted towards more acquisitive leaf traits (increasing leaf area, decreasing LDMC) and towards more conservative wood traits (increasing wood density) with chronic disturbance^42^. Cingolani et al.^43^ found, contrary to our findings, that sheep grazing shifted communities towards more acquisitive traits (high SLA) in Patagonian steppe grassland. Ultimately the effect of disturbance and stress on vegetation depends on the balance between resistance and recovery^44^: Grazing favours conservative, grazing-tolerant species when acquisitive, more palatable, species are eaten (creating resistant communities), on the other hand fire may increase acquisitive strategies with less flammable tissues whose fast growth allows quick recovery after a fire event (creating communities with a fast ability to recover) cf^45^. In addition, new recruits that colonize newly opened areas after a disturbance are most probably opportunistic pioneer species with acquisitive traits (communities with a fast ability to recover). This may also be what happens in severely eroded areas, instead of conservative species that resist the harsh and dry environments on eroded soil we found that these areas are colonized by opportunistic pioneer species with acquisitive leaf traits.

Dispersal limitation did not affect regenerative traits, unlike expected. Resprouting is a key trait that gives insight into the trade-off between regeneration from rootstock and from seedstock and is affected by disturbance regimes^46^. We included resprouting capacity as a binary trait but in reality it is a continuous trait where the proportion of biomass lost is related to the severity of disturbance and different categories and intensities of resprouting can be distinguished^19,46^. It is also a highly variable trait where the position along the gradient resprouting—seedling varies across a disturbance gradient, within and between species, across communities and even within individuals (eg with age)^46^. Since all our species had the potential to resprout we could not test this important regenerative strategy, further highlighting the unsuitability of resprouting capacity as a binary trait. In another study by the authors^47^, we inventoried regeneration mode across species in Kayoro (see Supplementary Fig. 6). Although it is not based in the same plots, and resprouting is highly context-specific, we see that both resprouting and regeneration from seed are common in the study region. To properly evaluate a site's regeneration potential it is crucial to do more detailed studies on modes of regeneration across different species and under different conditions of management, land degradation and proximity to seed sources. In terms of regenerative strategies, we found that grazing reduced seed mass. Small seeds are an indicator of an opportunistic recruitment strategy because small seeds have a low per capita investment and can travel large distances by wind thereby being able to colonize degraded areas^17^. This thus indicates recruitment of opportunistic seedlings entering the system under grazing. Shifts in functional properties of the regenerating communities composition suggest consequences for ecosystem functions (such as litter decomposition, productivity) and restoration benefits. Further studies on this are a research priority.

The 2021–2030 UN decade of ecosystem restoration raises high expectations of natural regeneration for restoration because it is considered cheap and effective. Studying landscape sites in West Africa we aimed to understand regeneration dynamics under different conditions of human impact, land degradation and dispersal limitation. Strong effects of dispersal limitation suggest that a significant proportion of natural regeneration comes from seed, while published work on FMNR mostly stresses the importance of regrowth from roots and stumps e.g.^11^, through 'releasing the underground forest'^48^. This realization suggests that the high tree densities in Sahelian agroforestry parklands, where FMNR has been demonstrated to work, are highly suitable for it to succeed but that scaling out beyond agroforestry parklands may lead to disappointing results. We found that this holds especially for the high value species *V. paradoxa* and the locally appreciated species *P. thonningii*. This makes it essential to assess regeneration potential before implementing FMNR. The proximity to forest reserves and availability of adult trees belonging to desired species is a precondition, while regeneration should be protected from grazing. When the regeneration potential is insufficient to achieve restoration targets, natural regeneration should be complemented with tree planting focusing on species important for ecosystem functioning and farmer livelihoods.

## Methods

### Study sites

This study takes place in two landscape sites in South-Centre Burkina Faso (Nobéré) and North-East Ghana (Navrongo), located about 60 km apart (see Supplementary Fig. 1). The study area is located in the Sudanian ecological zone and characterized by extensive farmland with median tree densities of about 30 trees per ha, commonly called agroforestry parklands^49^. The most common tree species is *Vitellaria paradoxa* (the shea tree), representing 38% of all trees and 12% of all regeneration (Supplementary Figs. 4 and 5). The annual rainfall is about 900 mm/yr and average annual temperature is 28 °C (Climate-data.org). Soils are classified as Lixisols, strongly weathered and leached soils with low nutrient status^37^. The landscapes consist of a mosaic of crop fields, fallows, grassland and forest or woodlands (see Supplementary Fig. 2). Agriculture is mostly subsistence-oriented in Burkina Faso with crops like yam, millet, sorghum and maize, while in Ghana besides subsistence crops also cotton is produced for the market. Farmers keep cows and goats and let them graze mainly on fallows and in forest or grassland during the cropping season and on crop residues inside the crop fields during the dry season^49^. In Burkina Faso stocking densities and grazing impact are higher than in Ghana (see Supplementary Fig. 3). Controlled fire in crop fields after harvesting may be used, though natural fire in fallows, bushland and forest is more common^50^. The use and intensity of fire is more common in Ghana than in Burkina Faso (see Supplementary Fig. 3). The two landscapes sites include six villages that are situated on the margin of protected forest areas (see Supplementary Fig. 1). Farmer-managed natural regeneration (FMNR) is a traditional practice across the region, though in Burkina Faso it has been actively promoted by environmental and development programs with the aim of restoring land and tree cover whilst improving local farmers' livelihoods.

### Sampling framework

The Land Degradation Surveillance Framework (LDSF) was used to assess management and biophysical indicators in the two landscape sites^51,52^. The LDSF uses a hierarchical sampling framework; each site is 100 km^2^, and consists of sixteen 1 km^2^ clusters, each cluster consists of ten randomly located 1000m^2^ sampling plots and each plot consists of four 100m^2^ subplots. In each plot all trees (DBH > 5 cm and height > 3 m) were identified and recorded, and in one smaller 20 m^2^ subplot in the middle of each plot all regeneration (DBH < 5 cm) was identified and recorded. In this study we used the plots (1,000 m^2^) as the unit of replication, resulting in 316 plots in total.

### Drivers of regeneration

#### Dispersal limitation

The distance of each plot to the nearest protected area (national park or forest reserve, see Supplementary Fig. 1) was calculated using the Rgeos Package in R^53^ and park locations and shapes were downloaded from protectedplanet.net. Further, we tested for the effect of the number of trees in the cluster (collection of 10 plots) and the number of trees in the plot. These three variables indicate measures of potential seed sources across different spatial scales (plot being smallest, then cluster, then closeness to reserve).

#### Land degradation

Land degradation was characterized by erosion and nitrogen content. The occurrence of erosion (0/1) was recorded in each of the four subplots that were summed to give a score of erosion severity at the plot level from 0 (no erosion) to 4 (severe erosion, present in all subplots). Topsoil samples (0–20 cm) were collected in each subplot, air-dried and thoroughly mixed to form a composite sample for each plot. About 1–2 g was ground to particle sizes of 20- 53 μm (RM 200 Retsch motor grinder) after which MIR absorbance was measured (Tensor 27 HTS-XT from Bruker Optics) at the World Agroforestry (ICRAF) Soil–Plant Spectral Diagnostics Laboratory in Nairobi, Kenya. Ten percent of the samples collected at each site (N = 16) were used as reference samples and analyzed for soil nitrogen content using dry combustion. Calibration models were developed for the prediction of soil properties using MIR spectra from the ICRAF pan-African MIR spectral library and validated using results of the soil analysis on the reference samples^54,55^. For this a random forest prediction model^56^ was fitted to derivatives computed using a Savitsky-Golay polynomial smoothing filter using the *KernSmooth* R package^57^, following^58^. Mid-infrared spectroscopy (MIRS) is becoming a well-established method for predicting soil properties^59–61^. The method used has been shown to accurately predict soil properties across sub-Saharan Africa^55^.

#### Human impact

The impact of agriculture, grazing and fire were each visually assessed at the plot level using a scale from 0 (no impact) to 3 (severe impact). Scoring was done by the same team for all plots in each site so although the method has some level of subjectivity, variation across the plots within each site are considered robust for analyses. See Supplementary Fig. 7 for photographs from the field illustrating the different human impact indicators and their scoring.

### Functional trait measurements and regenerating community properties

In total 50 species were recorded in the regeneration plots and 68 species in the tree plots, having a total of 86 species. Based on the abundance of regeneration across plots, 44 focal species were selected for functional trait measurements in the field. Eight functional traits (twig dry matter content, specific leaf area, leaf area, leaf dry matter content, leaf density, chlorophyll contents, chlorophyll fluorescence and leaf thickness) were measured on 5 adult individuals per species in the field, detailed methods of which are presented in the Supplementary information. Another eight traits (adult height, leaf phenology, ability to fix nitrogen, invasive properties, whether exotic, wood density, resprouting capacity and seed mass) were derived from literature sources. Supplementary Table 2 gives an overview of the traits used, their source and their functional significance. Species for which functional traits were available (either from the field or from the literature) cover well above 80% of the abundance across the plots (see Supplementary Table 3), which is considered to accurately represent the functional properties of the community^62^. The functional composition of regenerating communities was calculated using the community-weighted mean (CWM). The CWM represents the abundance-weighted average functional trait value of the community, calculated using the FD package^63^.

### Statistical analyses

We used the plot as the unit of observation, we have data for 316 plots, 160 for Seloghin (Burkina Faso) and 156 for Kayoro (Ghana). We characterized the regenerating community with 25 structural and functional variables (*structural variables*: seedling presence/ absence, number of seedlings, species richness of seedlings, presence and abundance of *Vitellaria paradoxa, Combretum glutinosum, Piliostigma thonningii*; *functional variables: community-weighted mean (CWM) of*; deciduousness (De), ability to fix N_2_, invasiveness, exotic, adult height, wood density (WD), twig dry matter content (TDMC), resprouting capacity, seed mass, specific leaf area (SLA), leaf area (LA), leaf dry matter contents (LDMC), leaf density (LD), chlorophyll content (Chl), leaf thickness (LT) and instantaneous chlorophyll fluorescence (Ft)).

We used generalised linear models (GLM) to test for the importance of drivers of regeneration, while including site as a fixed effect. The regeneration data is zero-inflated as 164 plots lacked regeneration. To this end our models follow a two-step approach. First we fit the model on the total dataset (N = 316) explaining the presence of regeneration as a binary variable (glm, family = binomial). Second we fit a series of models with the aim to understand the structural and functional characteristics of the regenerating community, based on a subset of the dataset that had seedlings present (N = 152). This resulted in a total of 25 GLMs. All statistical analyses were carried out in R version 3.3.2^64^.

### Informed consent

Informed consent has been obtained for publication of identifying images in an online open-access publication from the person identifiable in the picture in Box 1.

## Supplementary information

Supplementary Information.

## Data Availability

Data is available at https://data.worldagroforestry.org/dataset.xhtml?persistentId=doi:10.34725/DVN/RUFFFS.
