# Supplementary material for: Drivers of farmer-managed natural regeneration in the Sahel. Lessons for restoration
Source: Sci Rep. 2020 Sep 14;10:15038. doi: 10.1038/s41598-020-70746-z (PMC7490690; doi:10.1038/s41598-020-70746-z)
Supplement: Supplementary file 1 — Supplementary Information. [file 41598_2020_70746_MOESM1_ESM.pdf]

Supplementary information to:

Drivers of farmer-managed natural regeneration in the Sahel. Lessons for restoration

Madelon Lohbeck[1,2,*], Peggy Albers[2], Laetitia E. Boels[2], Frans Bongers[2], Samuel Morel[2], Fergus Sinclair[1,3], Bertin Takoutsing[4,5], Tor-Gunnar Vågen[1], Leigh A. Winowiecki[1], Emilie Smith-Dumont[1]

1 World Agroforestry (ICRAF), P.O. Box 30677-00100, Nairobi, Kenya

2 Forest Ecology and Forest Management Group, Wageningen University, P.O. Box 47, Wageningen, Netherlands

3 School of Natural Sciences, Bangor University, Wales, UK

4 World Agroforestry, P.O. Box 16317, Yaoundé, Cameroon

5 Soil Geography and Landscape Group, Wageningen University, PO Box 47, Wageningen

* correspondence to madelon.lohbeck@wur.nl

Supplementary Methods on functional traits measurement in the field

Supplementary Table 1. Summary of results of the effects of drivers land degradation tested with generalised linear models (GLMs)

Supplementary Table 2. Functional traits and their functional significance in the context of FMNR

Supplementary Table 3. Coverage of functional traits values across the regenerating communities

Supplementary Figure 1. Map of the study region

Supplementary Figure 2. Variation in variables studied across the main land uses in the region

Supplementary Figure 3. Variation in drivers of regeneration by site

Supplementary Figure 4. The number of trees for each species found across the study region

Supplementary Figure 5. The number of individuals regenerating for each species across the study region

Supplementary Figure 6. Regeneration mode across species; results from a separate study by the authors

Supplementary Figure 7. Illustrating human impact scores with photographs from the plots

Supplementary methods for functional traits measurement in the field

44 Focal species were selected for functional trait measurements. Eight traits (twig dry matter content, specific leaf area, leaf area, leaf dry matter content, leaf density, chlorophyll contents, chlorophyll fluorescence and leaf thickness) were measured on five adult individuals per species in the field. Standardized protocols for functional trait measurements were followed [1]. Plant material for trait measurements was sampled across the study region, not necessarily occurring inside the plots. Healthy individual trees were selected that had at least partially sun-exposed canopy. Three 20-30 cm long branches were cut from the tree, together having at least four fully expanded and sun-exposed leaves. Leaf chlorophyll content (SPAD units) was measured in the field on four selected leaves using a SPAD meter (Minolta SPAD-502, Spectrum Technologies, Plainfield, Illinois, USA). SPAD values correspond closely with independent measurements of chlorophyll contents [2]. Material was kept in plastic bags to limit desiccation. Soon after collection (max two hours) branches with the leaves attached were cut at the end and rehydrated in a bucket of water for at least one hour. Instantaneous chlorophyll fluorescence was measured on 4 leaves per branch with a fluorpen (Photon Systems Instruments, FP100) on exposure to 445 nm light at a saturating intensity of 3000 µE after dark adapting the leaves by covering them with a blanket inside a dark room. Four leaves (including the petiole, in case of compound species the entire leaf was taken) were selected and separated from the three branches per individual and one 20 cm twig per individual was selected. The area of each leaf (cm^2) was measured by photographing and analysing the photo using ImageJ software [3]. Leaf thickness (mm) was measured using a digital caliper, avoiding main veins. Fresh weight of each leaf was measured by weighting the leaf directly after rehydration, and the same was done for

the twig. Leaves and twigs were air-dried in labelled paper bags for several days until constant weight was achieved, and then weighted again for dry weight. Specific leaf area (m^2/kg) was calculated as the leaf area divided by its weight. Leaf dry matter content (g/g) was calculated as the dry weight divided by the fresh weight. Leaf density (g/cm^3) was calculated as the leaf's dry weight divided by its volume (thickness x area). Twig dry matter content (g/g) was calculated as the twig's dry weight divided by its fresh weight.

The other eight traits (adult height, leaf phenology, ability to fix nitrogen, invasive properties, whether exotic, wood density, resprouting capacity and seed mass) were derived from literature sources, see also Supplementary Table 2.

Supplementary Table 1. Summary of results of the effects of drivers of regeneration tested with generalised linear models. Each regeneration variable is a separate generalised linear model. Test statistics given are the standardized beta, the significance and the variance of the regeneration variable (Nagelkerke R^2) that was explained by the model. See also Figs 2 and 3 in the main manuscript for a graphical representation. Significant effects are underlined.

Regeneration ↓ / Drivers →	Human impact						Land degradation				Dispersal limitation								
	Agriculture		Grazing		Fire		Erosion		Nitrogen		Distance		Ntrees cluster		N trees plot		Site		
	β	P	β	P	β	P	β	P	β	P	β	P	β	P	β	P	β	P	R^2
presence/ absence [a]	-0.09	0.306	-0.26	0.045	0.17	0.078	0.04	0.498	0.01	0.874	-0.09	0.207	0.10	0.512	0.00	0.975	0.91	0.000	0.47
number of individuals [b]	-0.16	0.005	-0.18	0.027	0.16	0.003	0.13	0.004	-0.08	0.082	0.02	0.726	-0.05	0.346	0.07	0.239	1.61	0.000	0.65
Species richness	-0.16	0.093	0.11	0.483	0.15	0.170	0.10	0.193	-0.11	0.159	-0.23	0.014	-0.16	0.182	0.24	0.052	0.24	0.098	0.27
presence V. paradoxa	0.05	0.646	-0.46	0.031	0.22	0.069	0.13	0.160	-0.05	0.577	0.09	0.311	-0.11	0.372	0.21	0.017	0.76	0.000	0.21
abundance V. paradoxa	-0.07	0.636	-0.74	0.004	-0.03	0.864	0.12	0.349	-0.17	0.196	0.21	0.148	-0.37	0.046	0.27	0.019	1.57	0.006	0.14
presence C. glutinosum	-0.01	0.765	-0.07	0.108	0.00	0.898	0.02	0.406	0.04	0.102	0.04	0.051	-0.02	0.157	-0.15	0.999	1.04	0.988	0.40
abundance C. glutinosum	0.23	0.114	-0.32	0.197	0.21	0.197	0.20	0.150	0.27	0.022	0.37	0.009	-0.32	0.040	-1.92	0.996	18.79	0.984	0.38
presence P. thonningii	-0.01	0.941	0.13	0.345	-0.05	0.657	-0.01	0.884	-0.06	0.545	-0.15	0.078	-0.04	0.500	0.20	0.017	0.64	0.001	0.37
Structure: abundance P. thonningii	0.23	0.151	0.33	0.120	-0.05	0.782	0.02	0.893	-0.32	0.052	-0.28	0.107	0.01	0.949	0.31	0.000	2.11	0.054	0.37

Regeneration ↓ / Drivers →	Human impact						Land degradation				Dispersal limitation									
	Agriculture		Grazing		Fire		Erosion		Nitrogen		Distance		Ntrees cluster		N trees plot		Site		
	β	P	β	P	β	P	β	P	β	P	β	P	β	P	β	P	β	P	R^2
presence of C. nigricans	-0.22	0.045	0.05	0.477	-0.04	0.429	-0.03	0.514	-0.02	0.704	-0.09	0.071	0.01	0.568	0.02	0.393	0.96	0.993	0.51
abundance of C. nigricans	-1.35	0.007	-0.05	0.840	0.24	0.169	0.36	0.069	-0.53	0.041	-0.29	0.158	0.32	0.002	-0.04	0.619	17.46	0.986	0.58
presence of G. senegalensis	-0.11	0.008	0.12	0.042	0.03	0.366	0.06	0.163	-0.19	0.011	0.01	0.762	NA[d]	NA[d]	NA[d]	NA[d]	0.85	0.993	0.48
abundance of G. senegalensis	-0.76	0.001	0.37	0.223	0.40	0.045	1.13	0.000	-1.38	0.000	0.66	0.000	NA[d]	NA[d]	NA[d]	NA[d]	16.71	0.988	0.70

Regeneration ↓	Drivers →	Human impact						Land degradation				Dispersal limitation								
		Agriculture		Grazing		Fire		Erosion		Nitrogen		Distance		Ntrees cluster		N trees plot		Site		
		β	P	β	P	β	P	β	P	β	P	β	P	β	P	β	P	β	P	R^2
Whole-plant traits	CWM deciduous	0.15	0.173	-0.04	0.803	0.02	0.888	-0.06	0.511	0.05	0.556	-0.09	0.406	-0.28	0.028	-0.07	0.616	0.15	0.340	0.37
	CWM N_2 fix	0.03	0.818	-0.06	0.743	-0.09	0.474	0.07	0.453	-0.05	0.583	-0.17	0.131	0.01	0.960	0.03	0.849	-0.05	0.778	-0.03
	CWM invasive	-0.01	0.922	-0.04	0.800	-0.14	0.263	0.00	0.974	-0.06	0.499	0.05	0.662	-0.06	0.648	0.02	0.901	-0.02	0.883	-0.01
	CWM exotics	-0.01	0.916	0.02	0.909	-0.13	0.292	0.13	0.149	-0.06	0.498	0.09	0.374	0.05	0.708	-0.03	0.855	-0.16	0.327	-0.01
	CWM max height	-0.27	0.009	0.03	0.829	0.01	0.924	-0.14	0.095	-0.07	0.443	-0.12	0.229	-0.11	0.360	0.06	0.642	-0.36	0.017	0.22
	CWM WD	0.10	0.357	-0.31	0.073	-0.05	0.673	0.00	0.989	-0.11	0.234	0.04	0.704	-0.20	0.131	0.08	0.550	0.14	0.397	-0.05
	CWM TDMC	-0.17	0.117	0.27	0.107	-0.12	0.298	0.00	0.983	0.12	0.193	-0.19	0.068	-0.10	0.441	0.08	0.533	-0.39	0.014	-0.01
Regenerative traits	CWM resprouting[c]	NA[c]	NA[c]	NA[c]	NA[c]	NA[c]	NA[c]	NA[c]	NA[c]	NA[c]	NA[c]	NA[c]	NA[c]	NA[c]	NA[c]	NA[c]	NA[c]	NA[c]	NA[c]	NA[c]
	CWM seed mass	0.07	0.515	-0.41	0.023	0.10	0.397	-0.04	0.628	-0.14	0.131	0.15	0.177	-0.18	0.171	0.03	0.836	0.19	0.251	0.11
Acquisitive leaf traits	CWM SLA	-0.18	0.078	-0.05	0.778	-0.01	0.951	-0.20	0.020	0.03	0.727	-0.12	0.227	-0.13	0.277	0.19	0.144	-0.24	0.111	0.20
	CWM leaf area	0.04	0.741	-0.02	0.898	-0.06	0.616	0.20	0.035	-0.09	0.350	-0.02	0.818	-0.09	0.482	0.06	0.651	-0.17	0.312	-0.00
	CWM Ft	-0.19	0.051	0.08	0.597	-0.05	0.636	-0.08	0.361	0.09	0.286	0.04	0.689	0.12	0.303	0.14	0.254	-0.52	0.000	0.29
	CWM Chl	-0.05	0.652	0.20	0.257	-0.13	0.277	0.15	0.114	0.01	0.879	0.12	0.262	0.20	0.134	-0.01	0.915	-0.24	0.135	0.07
Conservative leaf traits	CWM LDMC	-0.13	0.215	0.36	0.030	-0.25	0.032	0.12	0.195	0.07	0.422	-0.09	0.394	-0.03	0.804	0.07	0.588	-0.11	0.470	-0.01
	CWM LD	-0.19	0.103	0.13	0.475	-0.04	0.731	-0.01	0.956	-0.03	0.730	-0.05	0.637	-0.06	0.653	0.03	0.842	0.02	0.894	-0.05
	CWM LT	0.25	0.021	0.08	0.617	-0.06	0.586	0.08	0.343	0.01	0.915	-0.08	0.447	0.09	0.481	-0.22	0.093	0.11	0.484	-0.06

[a] binomial
[b] poisson
[c] all species encountered have the ability to resprout so there was no variation in the data to be able to test
[d] no trees were found of *G. senegalensis* so these variables could not be tested

Supplementary Table 2. Functional traits used in this study, data source, main environmental filter it responds to, functional significance in the context of FMNR and some key references

Trait category	Functional trait	Data source	Main env. filter	Functional significance	References
whole plant trait	Adult height (m)	Literature, field observations	Disturbance, farmers preferences	Aboveground biomass, competition for light, economic value	[4,5]
whole plant trait	Leaf phenology (0 = evergreen, 1 = deciduous)	Literature, field observations	Stress, disturbance	Drought avoidance, evapotranspiration, leaf longevity, decomposition, litter production	[6–8]
whole plant trait	Nitrogen fixing (0/1)	Literature, field observations	Stress, disturbance, farmers preferences	Growth rate, foliar nitrogen, N-mineralization, soil nitrification, water-use efficiency	[9–12]
whole plant trait	Invasive (0/1)	Literature	Stress, disturbance, farmers preferences	Outcompete original vegetation, fast growth and reproduction	[13–15]
whole plant trait	Exotic (0/1)	Literature	Farmers preferences	Species not naturally occurring in area but often promoted for specific properties	
whole plant trait	Wood density (g/cm3)	Literature	Farmer preferences	Construction costs, growth rate, stem vulnerability, mortality rate	[16–18]
whole plant trait	Twig dry matter content (g/g)	Field measurements	Stress, disturbance	Water storage, drought resistance, flammability, mortality rate	[19]
regenerative trait	Resprouting capacity (0/1)	Field observations, local informants, literature	Environmental drivers, stress, disturbance	Drought avoidance, resistance to fire and herbivory	[20–23]
regenerative trait	Seed mass; SV (g)	Literature	Environmental drivers, stress, disturbance	Investment in reproduction, germination success, number of seeds, photosynthetic rates of seedlings, distance of dispersal, colonization chance, longevity in seed bank	[24–29]

acquisitive leaf trait	Specific leaf area; SLA (m2/g)	Field measurements	Stress, disturbance	Light capture economics, net assimilation rate, relative growth rate, leaf life span, photosynthetic capacity	[30–32]
acquisitive leaf trait	Leaf area; LA (cm^2)	Field measurements	Stress, disturbance	Light intercepting area, respiration, transpiration, leaf cooling, gas exchange	[33–35]
acquisitive leaf trait	Chlorophyll content; Chl (SPAD-units)	Field measurements	Stress, disturbance	Light uptake efficiency, photosynthetic rates	[31,36,37]
acquisitive leaf trait	Instantaneous chlorophyll fluorescence; F_0 (no unit)	Field measurements	Stress, disturbance	Heat dissipation, resistance against overheating, photosynthetic performance	[38–40]
conservative leaf trait	Leaf Dry Matter Content; LDMC ($g\ g^{-1}$)	Field measurements	Stress, disturbance	Construction costs, nutrient retention, against mechanical and herbivore damage, drought tolerance, flammability	[41–43]
conservative leaf trait	Leaf Density; LD ($g\ cm^{-3}$)	Field measurements	Stress, disturbance	Construction costs, leaf life span, photosynthetic rates per unit leaf area, against mechanical and herbivore damage	[44,45]
conservative leaf trait	Leaf thickness; LT (mm)	Field measurements	Stress, disturbance	Construction costs, leaf life span, photosynthetic rates per unit leaf area, against mechanical and herbivore damage, gas exchange and leaf cooling	[34,42,46,47]

Supplementary Table 3. The coverage of functional traits across all regenerating communities when weighted by number of individuals. For the community functional properties to be representative for the whole community, a trait coverage of at least 0.8 has been suggested [48]

**Trait category**	**Functional trait**	**Median**	**Mean**	**SD**
whole plant trait	Max height (m)	1.00	0.81	0.30
whole plant trait	Leaf Phenology (0/1)	1.00	0.91	0.23
whole plant trait	Nitrogen fixing (0/1)	1.00	0.90	0.23
whole plant trait	Invasive (0/1)	1.00	1.00	0.00
whole plant trait	Exotic (0/1)	1.00	0.99	0.08
whole plant trait	TDMC (g/g)	1.00	0.87	0.27
whole plant trait	Wood density (g/cm3)	1.00	0.92	0.21
regenerative trait	Resprouting capacity (0/1)	1.00	0.99	0.11
regenerative trait	seed mass (g)	1.00	0.79	0.31
acquisitive leaf trait	SLA (m2/g)	1.00	0.87	0.27
acquisitive leaf trait	LA (m2)	1.00	0.87	0.27
acquisitive leaf trait	Chl (SPAD units)	1.00	0.87	0.27
acquisitive leaf trait	Ft (no unit)	1.00	0.85	0.28
conservative leaf trait	LDMC (g/g)	1.00	0.87	0.27
conservative leaf trait	LD (g/cm3)	1.00	0.81	0.31
conservative leaf trait	LT (mm)	1.00	0.81	0.31

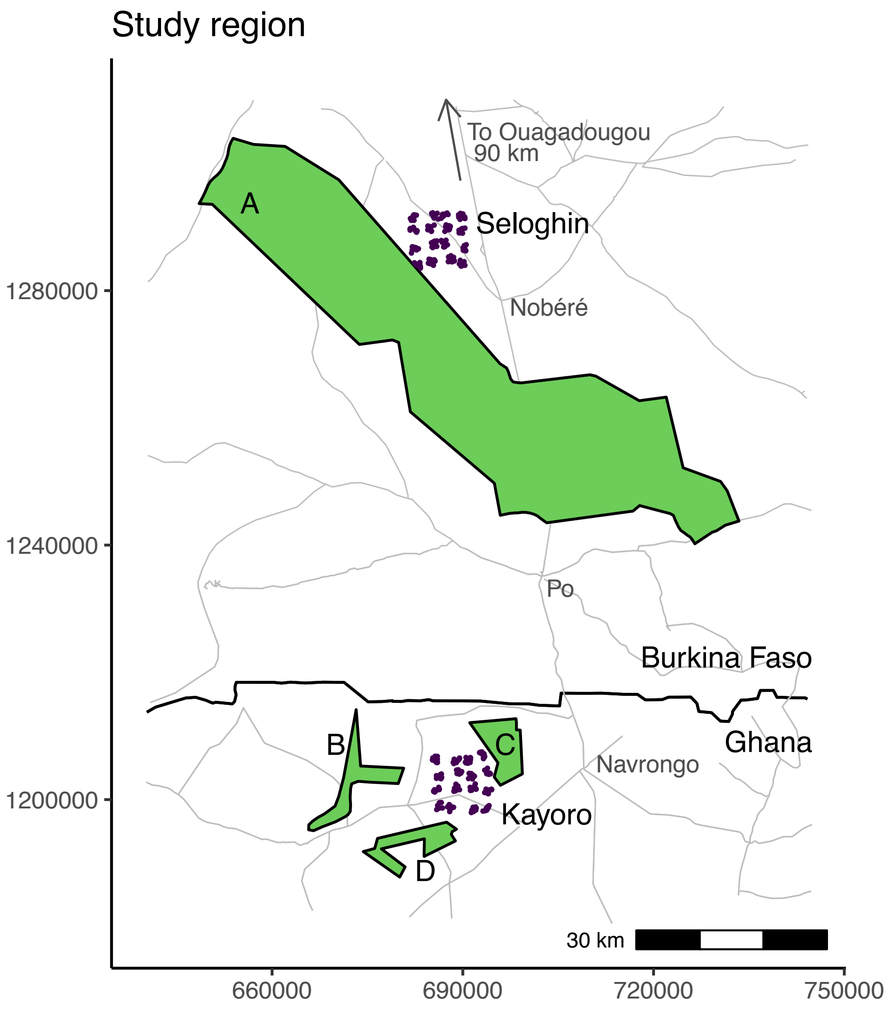

Supplementary Figure 1. Map of the study region with the Seloghin LDSF site in South Burkina Faso and the Kayoro LDSF site in North East Ghana, each site having approximately 160 plots. Both sites are close to protected areas, being; A) Kaboré Tambi national park, B) Chiana hills forest reserve, C) Chasi river forest reserve and D) Bopong forest reserve (www.protectedplanet.org). Projection: WGS84 UTM Zone 30.

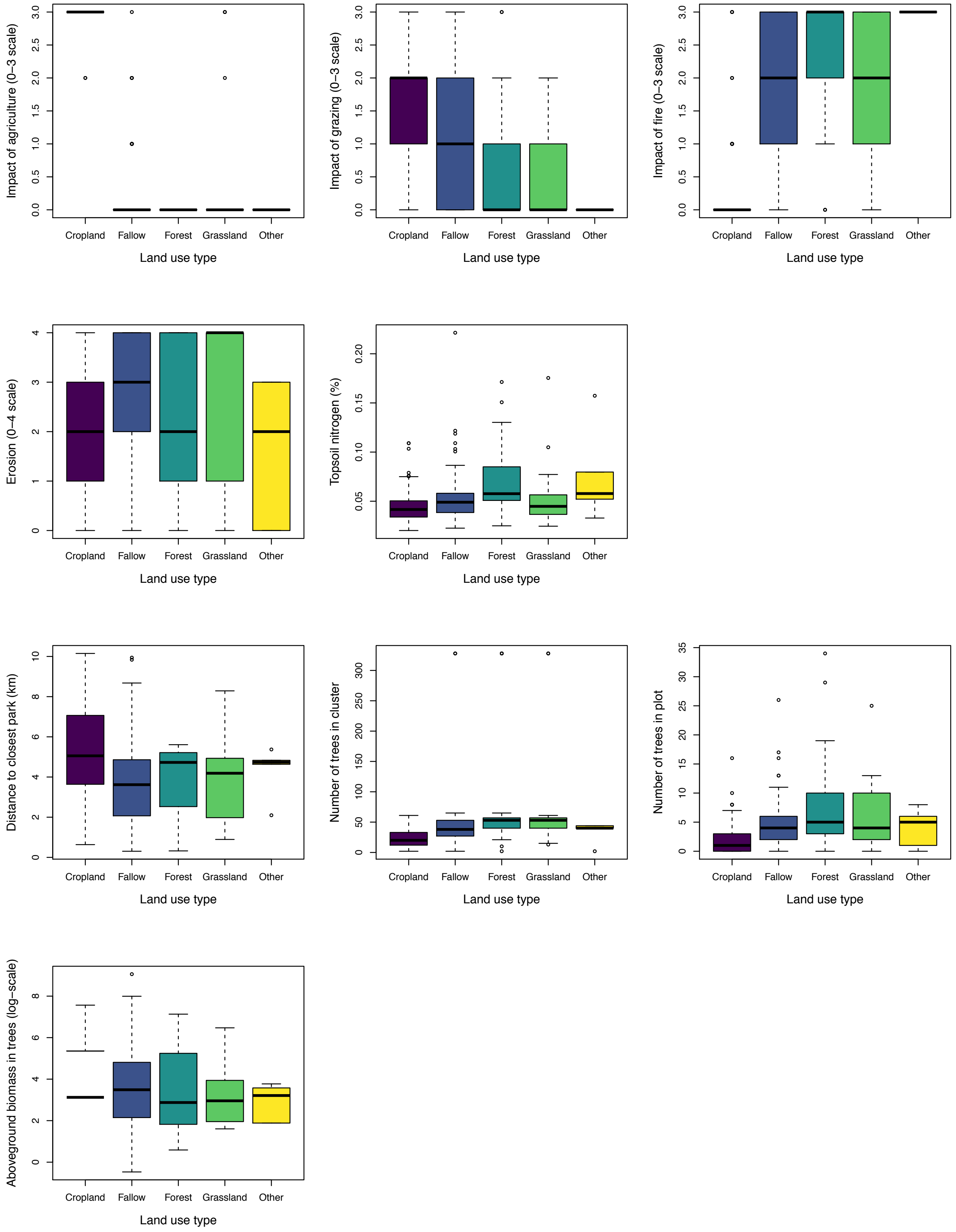
Impact of agriculture (0–3 scale)
Impact of grazing (0–3 scale)
Impact of fire (0–3 scale)
Erosion (0–4 scale)
Topsoil nitrogen (%)
Distance to closest park (km)
Number of trees in cluster
Number of trees in plot
Aboveground biomass in trees (log–scale)
Cropland
Fallow
Forest
Grassland
Other
Land use type

20

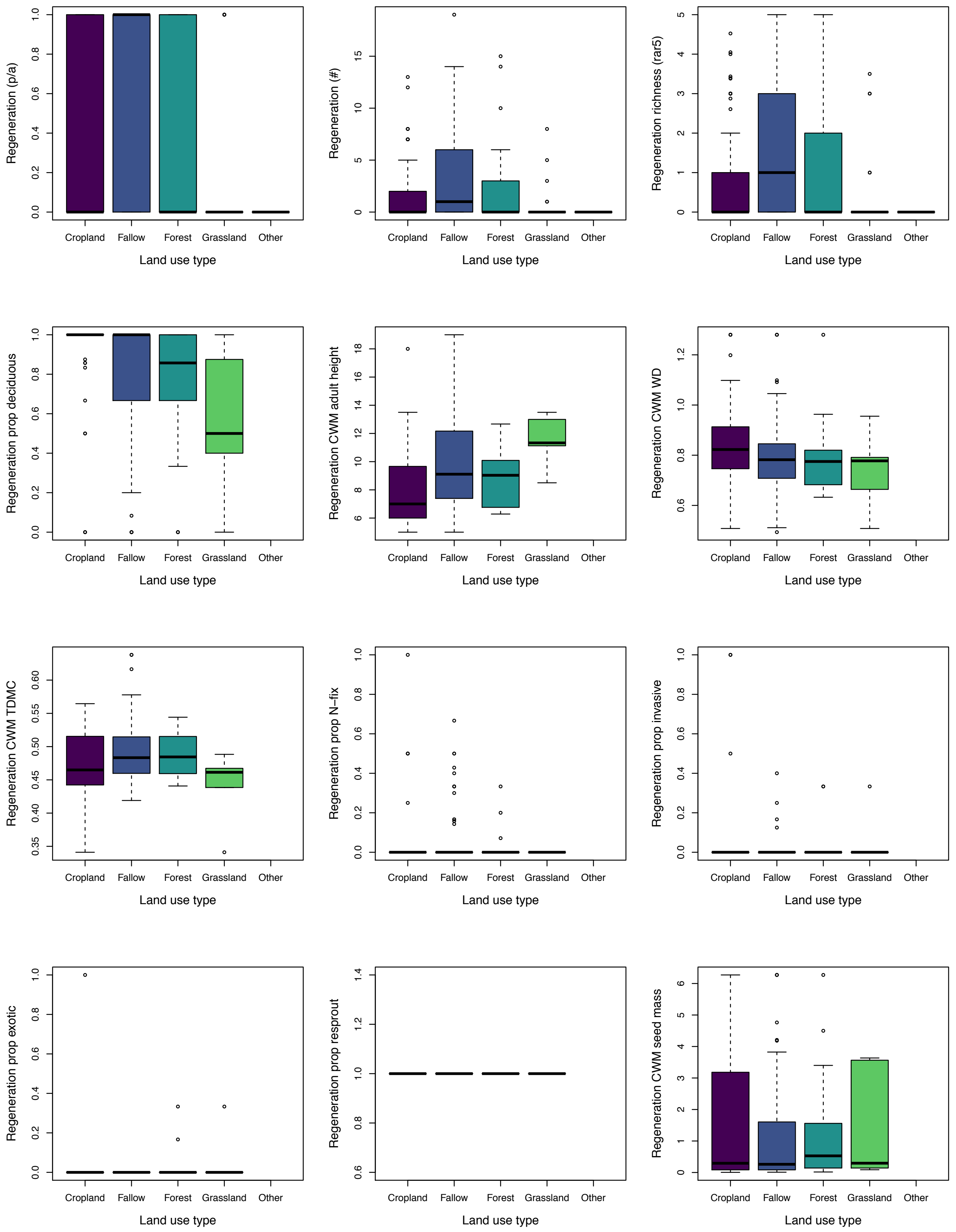
Regeneration (p/a)
Regeneration (#)
Regeneration richness (rar5)
Regeneration prop deciduous
Regeneration CWM adult height
Regeneration CWM WD
Regeneration CWM TDMC
Regeneration prop N−fix
Regeneration prop invasive
Regeneration prop exotic
Regeneration prop resprout
Regeneration CWM seed mass
Cropland
Fallow
Forest
Grassland
Other
Land use type

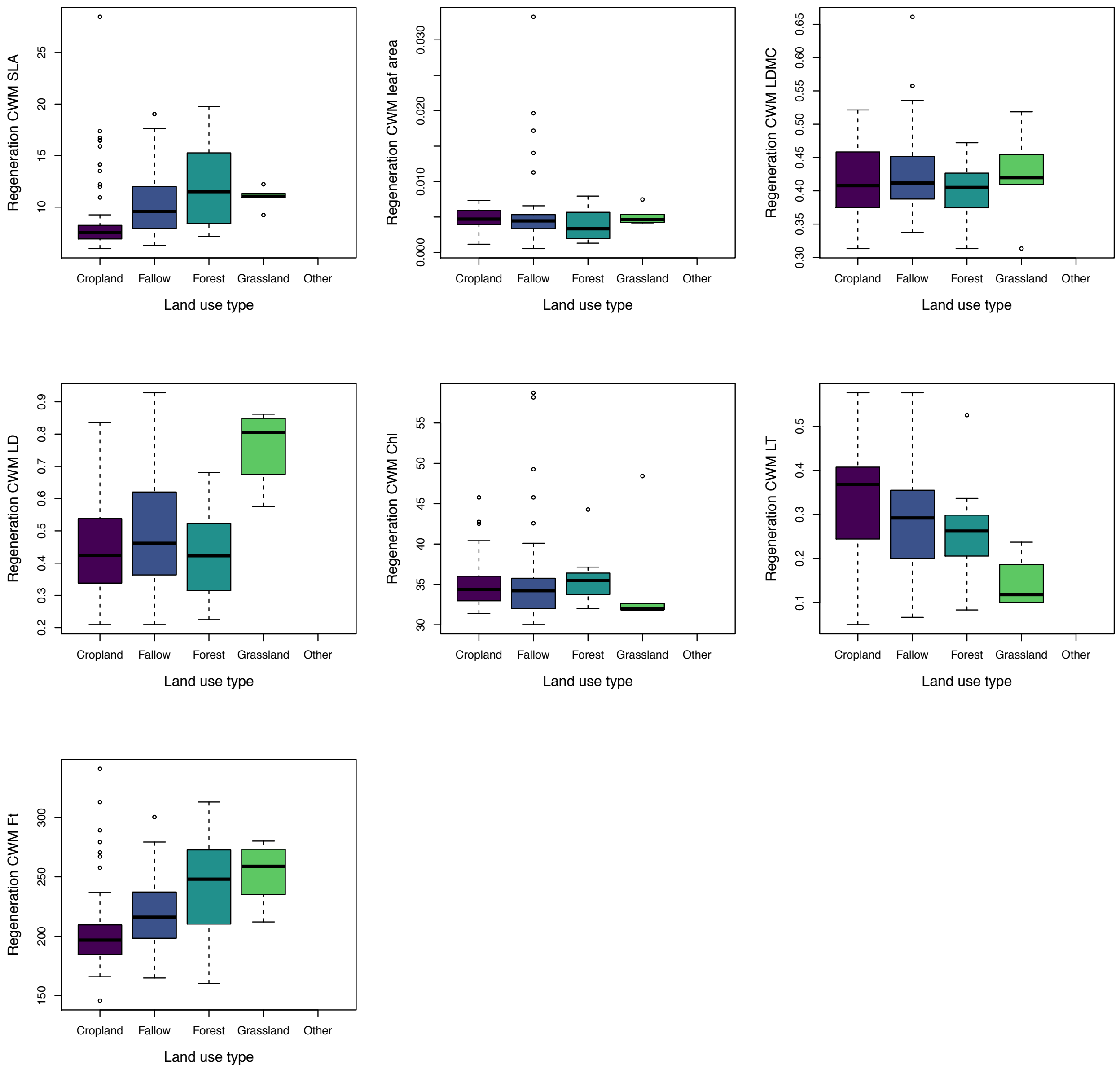

Supplementary Figure 2. All variables studied against the main land use types in the region (cropland N = 129, fallow N = 129, forest N = 41, grassland N = 21, other N = 5)

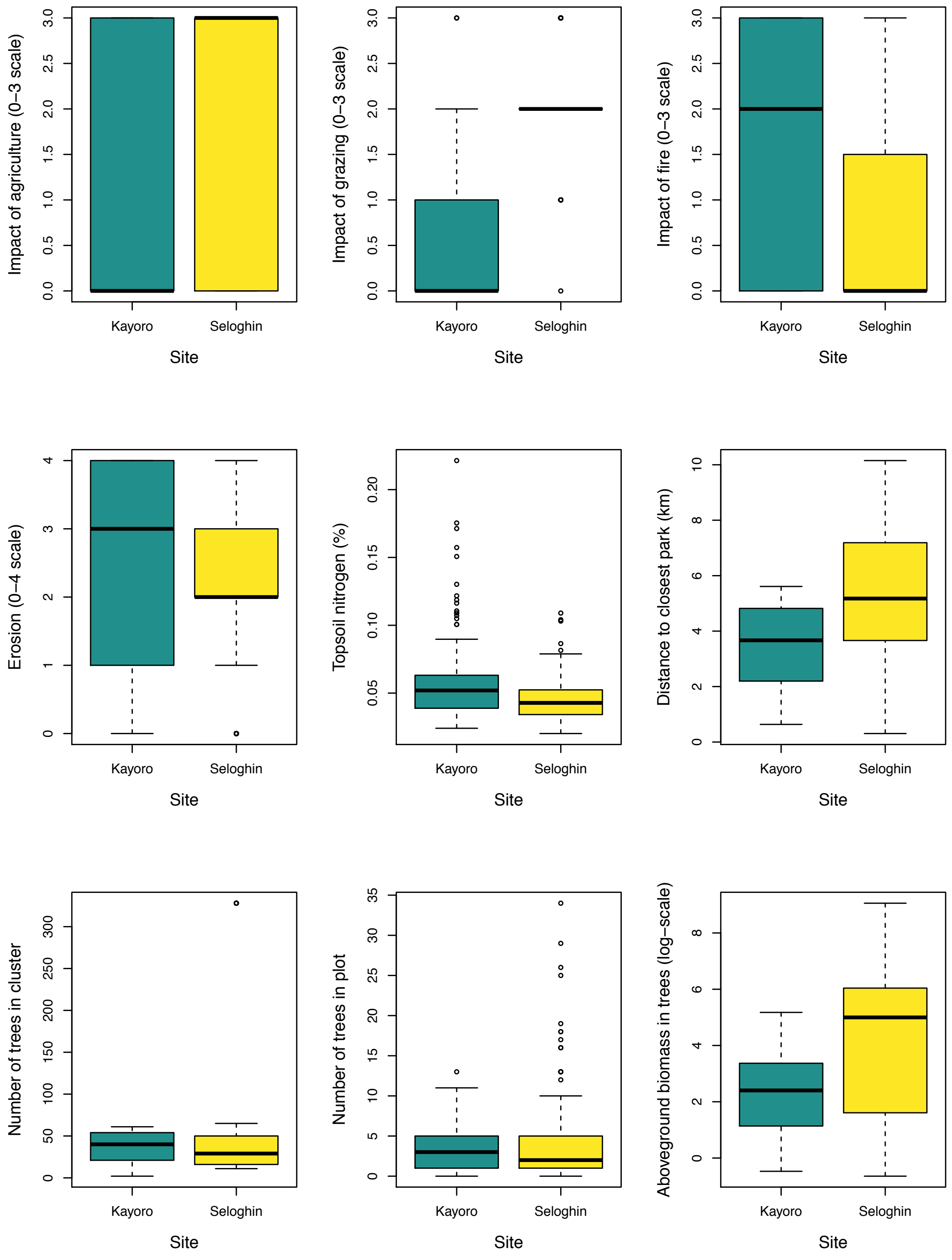

Supplementary Figure 3. Variation in drivers of regeneration by site (Kayoro in Ghana N = 156, Seloghin in Burkina Faso N = 160)

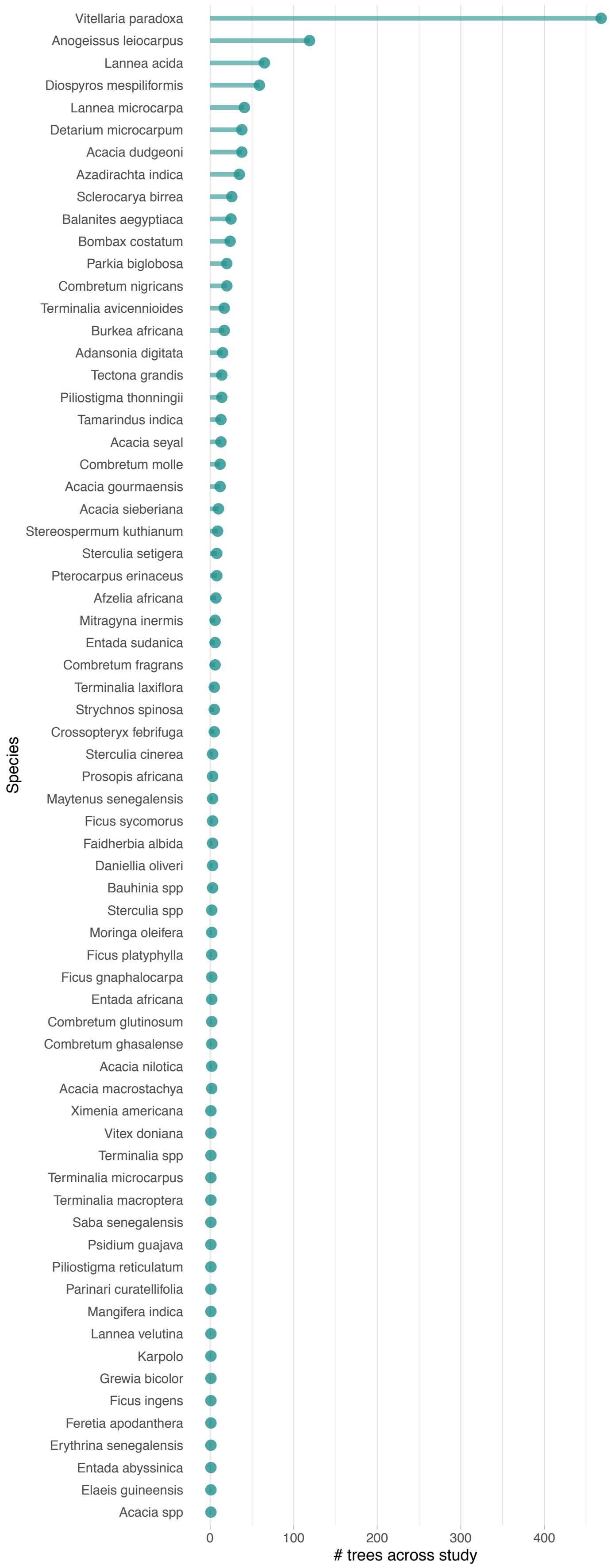

31 Supplementary Figure 4. The number of trees for each species across the study region

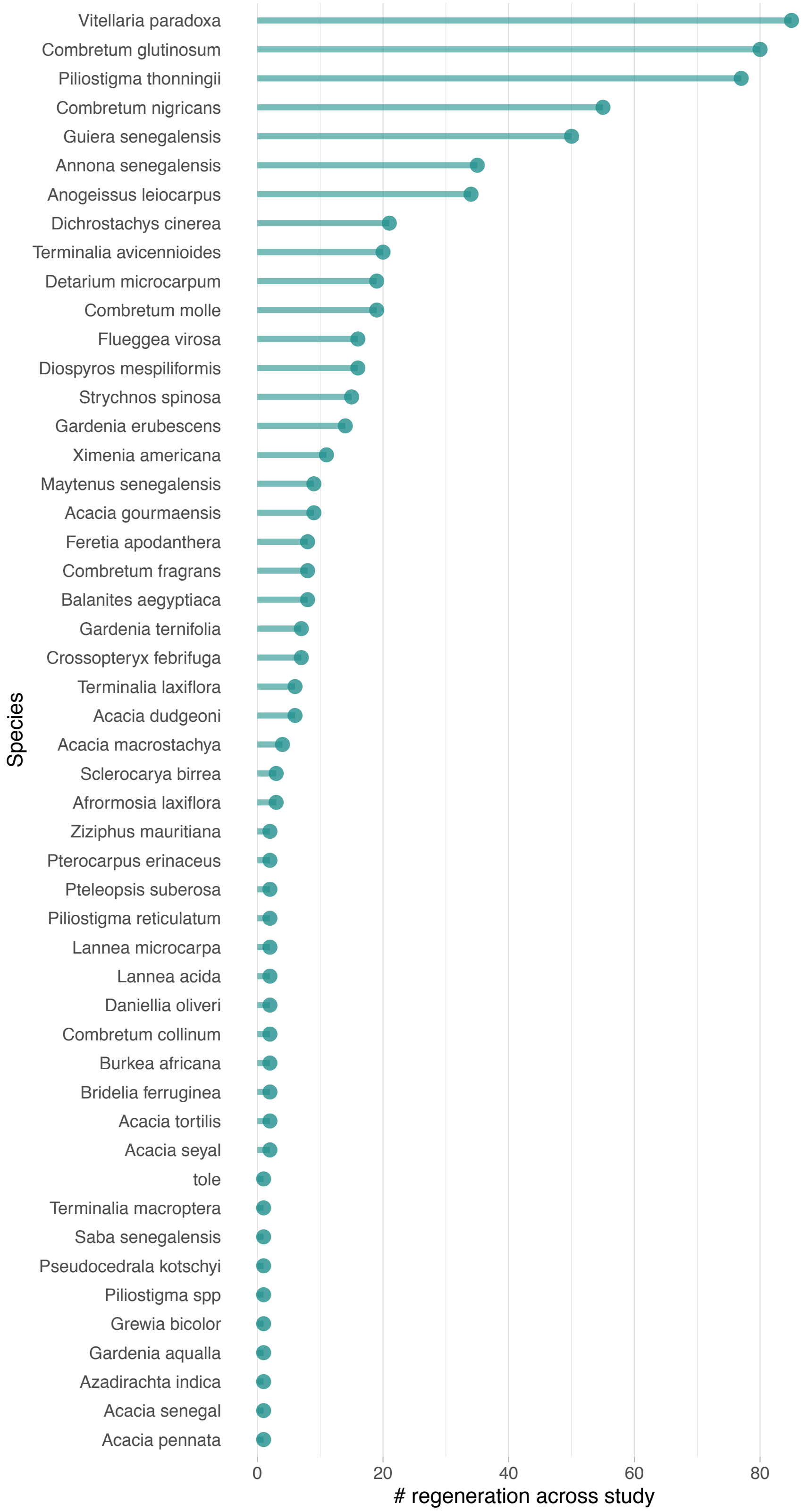

Supplementary Figure 5. The number of regenerating individuals for each species across the study region

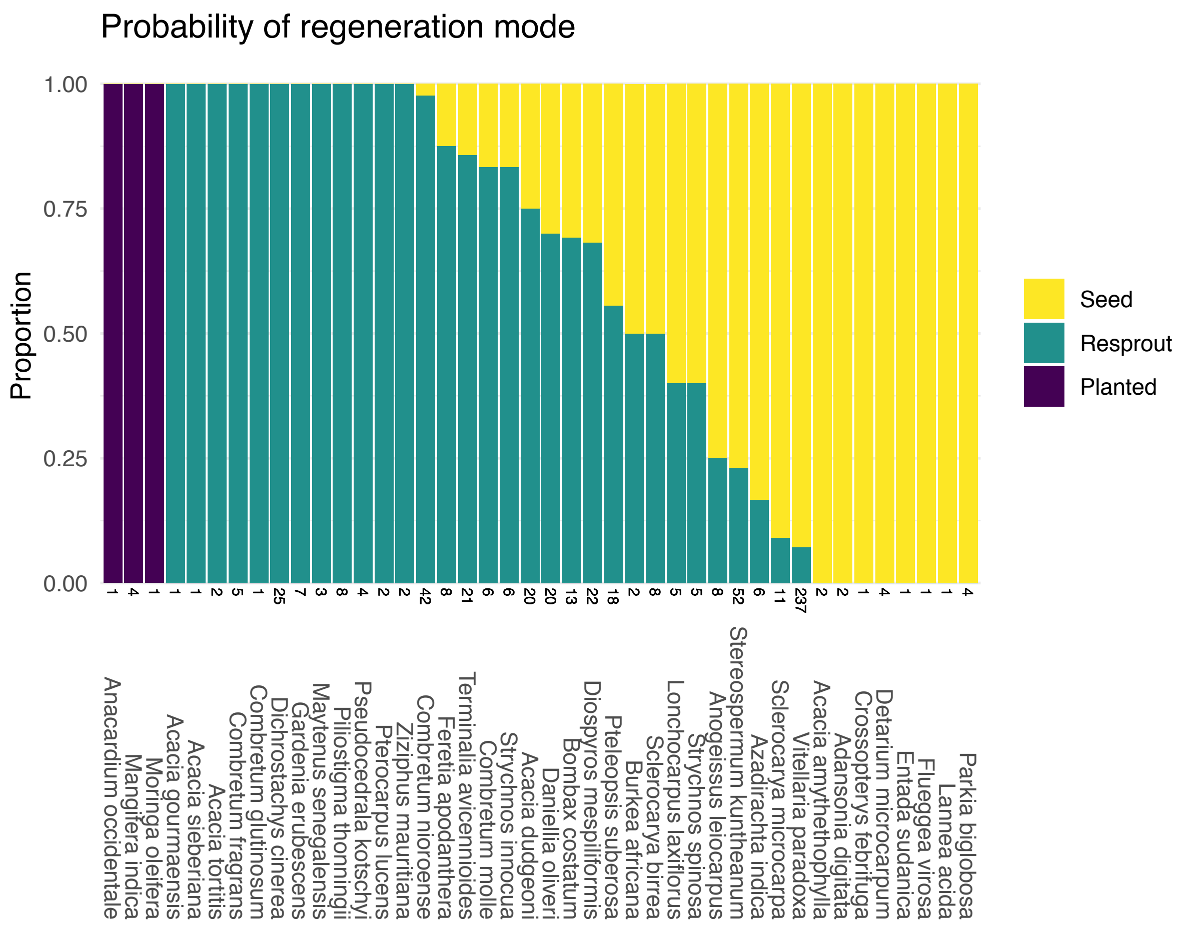

Supplementary Figure 6. Regeneration mode across species; results from a separate study by the authors [49], based on 40 (different) plots in the same site Kayoro, Ghana. Regeneration inventories were made and for each individual the regeneration mode was assessed based on observations in the field and farmers' knowledge. The numbers below the bars represent the total numbers of individuals found for that species.

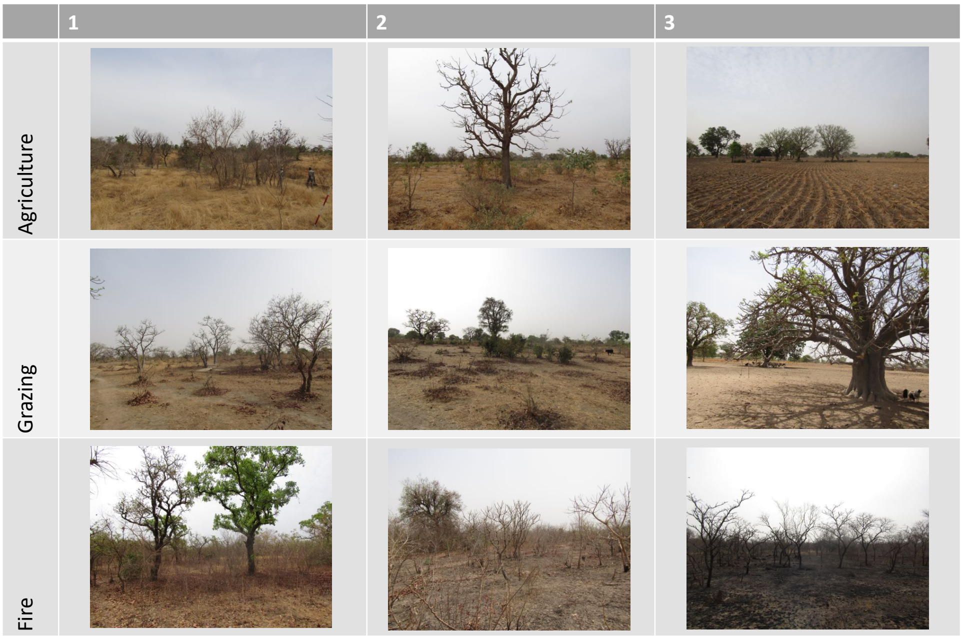

Supplementary Figure 7. Illustrating human impact scores with photographs from the plots in Seloghin in South Burkina Faso. Different impact indicators that are used as drivers in this study (agriculture, grazing, fire) are given as rows and the scores from 1 (light impact) to 3 (severe impact) are given as columns. Score of 0 (no impact) is not shown. Photographs by François Kagambèga Wenemi.

Bibliography to supplementary materials

1. Pérez-Harguindeguy, N. *et al.* New handbook for standardised measurement of plant functional traits worldwide. *Aust. J. Bot.* **61**, 167–234 (2013).
2. Coste, S., Baraloto, C., Leroy, C. & Marcon, E. Assessing foliar chlorophyll contents with the SPAD-502 chlorophyll meter: a calibration test with thirteen tree species of tropical rainforest in French Guiana. *Ann. For. Sci.* (2010).
3. Rasband, W. S. ImageJ. (2008).
4. Chapin, F. S. Effects of plant traits on ecosystem and regional processes: a conceptual framework for predicting the consequences of global change. *Ann. Bot.* **91**, 455–463 (2003).
5. Lin, D. *et al.* Traits of dominant tree species predict local scale variation in forest aboveground and topsoil carbon stocks. *Plant Soil* **409**, 435–446 (2016).
6. Hacke, U. G., Sperry, J. S. & Pittermann, J. Drought experience and cavitation resistance in six shrubs from the Great Basin, Utah. *Basic Appl. Ecol.* **1**, 31–41 (2000).
7. Stokes, A., Atger, C., Bengough, A. G., Fourcaud, T. & Sidle, R. C. Desirable plant root traits for protecting natural and engineered slopes against landslides. *Plant Soil* **324**, 1–30 (2009).
8. Zanne, A. E. *et al.* Data from: Three keys to the radiation of angiosperms into freezing environments. *Dryad Digit. Repos.* (2013). doi:10.5061/dryad.63q27.2
9. Siddique, I. *et al.* Dominance of legume trees alters nutrient relations in mixed species forest restoration plantings within seven years. *Biogeochemistry* **88**, 89–101 (2008).
10. Gei, M. G. & Powers, J. S. Do legumes and non-legumes tree species affect soil properties in unmanaged forests and plantations in Costa Rican dry forests? *Soil Biol.*

Biochem. **57**, 264–272 (2013).
11. Doland Nichols, J., Rosemeyer, M. E., Carpenter, F. L. & Kettler, J. Intercropping legume trees with native timber trees rapidly restores cover to eroded tropical pasture without fertilization. *For. Ecol. Manage.* **152**, 195–209 (2001).
12. Adams, M. A., Turnbull, T. L., Sprent, J. I. & Buchmann, N. Legumes are different: Leaf nitrogen, photosynthesis, and water use efficiency. *PNAS* **113**, 4098–4103 (2016).
13. Jackson, R. B., Banner, J. L., Jobbágy, E. G., Pockman, W. T. & Wall, D. H. Ecosystem carbon loss with woody plant invasion of grasslands. *Nature* **418**, 620–623 (2002).
14. Vågen, T.-G. & Winowiecki, L. *Northern Rangelands Trust. Baseline assessment of rangeland health, Kalama and Namunyak conservancies*. (ICRAF, CIAT, 2014).
15. IUCN. Global Invasive Species Database. http://www.iucngisd.org/gisd/ (2019).
16. Zanne, A. E. *et al.* Data from: Towards a worldwide wood economics spectrum. *Dryad Data Repos.* (2009). doi:10.5061/dryad.234
17. Augspurger, C. K. & Kelly, C. K. Pathogen mortality of tropical tree seedlings: Experimental studies of the effects of dispersal distance, seedling density, and light conditions. *Oecologia* **61**, 211–217 (1984).
18. Chave, J. *et al.* Towards a worldwide wood economics spectrum. *Ecol. Lett.* **12**, 351–366 (2009).
19. Cornelissen, J. H. C. *et al.* A handbook of protocols for standardised and easy measurement of plant functional traits worldwide. *Aust. J. Bot.* **51**, 335–380 (2003).
20. Paula, S. & Pausas, J. G. Leaf traits and resprouting ability in the Mediterranean basin. *Funct. Ecol.* **20**, 941–947 (2006).
21. Bond, W. J. & Midgley, J. J. Ecology of sprouting in woody plants: the persistence niche. *Trends Ecol Evol* **16**, 45–51 (2001).

22. Bellefontaine, R. Synthèse des espèces des domaines sahélien et soudanien qui se multiplient naturellement par voie végétative. in *Fonctionnement et gestion des écosystèmes forestiers contractés sahéliens* (eds. D'Herbès, L., Ambouta, L. K. & Peltier., R.) (John Libbey Eurotext, Paris, 1997).

23. Ky-Dembele, C., Tigabu, M., Bayala, J., Ouédraogo, S. J. & Odén, P. C. The relative importance of different regeneration mechanisms in a selectively cut savanna-woodland in Burkina Faso, West Africa. *For. Ecol. Manage.* **243**, 28–38 (2007).

24. Foster, S. & Janson, C. H. The relationship between seed size and establishment conditions in tropical woody plants. *Ecology* **66**, 773–780 (1985).

25. Kitajima, K. Relationship between photosynthesis and thickness of cotyledons for tropical forest tree species. *Funct. Ecol.* **6**, 582–589 (1992).

26. Westoby, M., Leishman, M., Lord, J., Poorter, H. & Schoen, D. J. Comparative ecology of seed size and dispersal [and discussion]. *Philos. Trans. R. Soc. London. Ser. B Biol. Sci.* **351**, 1309–1318 (1996).

27. Weiher, E. *et al.* Challenging Theophrastus: A common core list of plant traits for functional ecology. *J. Veg. Sci.* **10**, 609–620 (1999).

28. Moles, A. T. & Westoby, M. Seedling survival and seed size: a synthesis of the literature. *J. Ecol.* **92**, 372–383 (2004).

29. Kew Royal Botanic Gardens. Seed Information Database (SID). .(2019) Version 7.1. Available from: http://data.k (2019).

30. Cornelissen, J. H. C. *et al.* Leaf structure and defence control litter decomposition rate across species and life forms in regional floras on two continents. *New Phytol.* **143**, 191–200 (1999).

31. Poorter, L. & Bongers, F. Leaf traits are good predictors of plant performance across

53 rain forest species. *Ecology* **87**, 1733–1743 (2006).

32. Poorter, H., Niinemets, Ü., Poorter, L., Wright, I. J. & Villar, R. Causes and consequences of variation in leaf mass per area (LMA): a meta-analysis. *New Phytol.* **182**, 565–588 (2009).

33. Bazzaz, F. A. & Pickett, S. T. A. Physiological ecology of tropical succession: A comparative review. *Annu. Rev. Ecol. Syst.* **11**, 287–310 (1980).

34. Popma, J., Bongers, F. & Werger, M. J. A. Gap-dependence and leaf characteristics of trees in a tropical lowland rain forest in Mexico. *Oikos* **63**, 207–214 (1992).

35. Yates, M. J., Anthony Verboom, G., Rebelo, A. G. & Cramer, M. D. Ecophysiological significance of leaf size variation in Proteaceae from the Cape Floristic Region. *Funct. Ecol.* **24**, 485–492 (2010).

36. Minotta, G. & Pinzauti, S. Effects of light and soil fertility on growth, leaf chlorophyll content and nutrient use efficiency of beech (Fagus sylvatica L.) seedlings. *For. Ecol. Manage.* **86**, 61–71 (1996).

37. Rozendaal, D. M. A., Hurtado, V. H. & Poorter, L. Plasticity in leaf traits of 38 tropical tree species in response to light; relationships with light demand and adult stature. *Funct. Ecol.* **20**, 207–216 (2006).

38. Maxwell, K. & Johnson, G. N. Chlorophyll fluorescence-a practical guide. *J. Exp. Bot.* **51**, 659–668 (2000).

39. Baker, N. R. Applications of chlorophyll fluorescence can improve crop production strategies: an examination of future possibilities. *J. Exp. Bot.* **55**, 1607–1621 (2004).

40. Woo, N. S., Badger, M. R. & Pogson, B. J. A rapid, non-invasive procedure for quantitative assessment of drought survival using chlorophyll fluorescence. *Plant Methods* **4**, 27 (2008).

41. Poorter, H. & Garnier, E. Ecological significance of relative growth rate and its components. in *Handbook of Functional Plant Ecology* (eds. Pugnaire, F. I. & Valladares, F.) 81–120 (Marcel Dekker, 1999).
42. Niinemets, Ü. Global-scale climatic controls of leaf dry mass per area, density, and thickness in trees and shrubs. *Ecology* **82**, 453–469 (2001).
43. Garnier, E. *et al.* Plant functional markers capture ecosystem properties during secondary succession. *Ecology* **85**, 2630–2637 (2004).
44. Niinemets, Ül. Research review. Components of leaf dry mass per area – thickness and density – alter leaf photosynthetic capacity in reverse directions in woody plants. *New Phytol.* **144**, 35–47 (1999).
45. Lebrija-Trejos, E., Pérez-García, E. A., Meave, J. A., Bongers, F. & Poorter, L. Functional traits and environmental filtering drive community assembly in a species-rich tropical system. *Ecology* **91**, 386–398 (2010).
46. Reich, P. B., Uhl, C., Walters, M. B. & Ellsworth, D. S. Leaf lifespan as a determinant of leaf structure and function among 23 amazonian tree species. *Oecologia* **86**, 16–24 (1991).
47. Onoda, Y. *et al.* Global patterns of leaf mechanical properties. *Ecol. Lett.* **14**, 301–312 (2011).
48. Pakeman, R. J. & Quested, H. M. Sampling plant functional traits: What proportion of the species need to be measured? *Appl. Veg. Sci.* **10**, 91–96 (2007).
49. Albers, P. Linking household strategies to natural regeneration in West African parklands. (MSc thesis Wageningen University, 2019).
